# Genetic Variation of an Odorant Receptor OR7D4 and Sensory Perception of Cooked Meat Containing Androstenone

**DOI:** 10.1371/journal.pone.0035259

**Published:** 2012-05-02

**Authors:** Kathrine Lunde, Bjørg Egelandsdal, Ellen Skuterud, Joel D. Mainland, Tor Lea, Margrethe Hersleth, Hiroaki Matsunami

**Affiliations:** 1 Norwegian Meat Research Centre, Oslo, Norway; 2 Institute of Chemistry, Biotechnology and Food Science, University of Life Science, Ås, Norway; 3 Nofima Mat, Ås, Norway; 4 Department of Molecular Genetics and Microbiology, and Neurobiology, Duke University Medical Center, Durham, North Carolina, United States of America; German Institute of Human Nutrition Potsdam-Rehbruecke, Germany

## Abstract

Although odour perception impacts food preferences, the effect of genotypic variation of odorant receptors (ORs) on the sensory perception of food is unclear. Human OR7D4 responds to androstenone, and genotypic variation in *OR7D4* predicts variation in the perception of androstenone. Since androstenone is naturally present in meat derived from male pigs, we asked whether OR7D4 genotype correlates with either the ability to detect androstenone or the evaluation of cooked pork tainted with varying levels of androstenone within the naturally-occurring range. Consistent with previous findings, subjects with two copies of the functional OR7D4 RT variant were more sensitive to androstenone than subjects carrying a non-functional OR7D4 WM variant. When pork containing varying levels of androstenone was cooked and tested by sniffing and tasting, subjects with two copies of the RT variant tended to rate the androstenone-containing meat as less favourable than subjects carrying the WM variant. Our data is consistent with the idea that OR7D4 genotype predicts the sensory perception of meat containing androstenone and that genetic variation in an odorant receptor can alter food preferences.

## Introduction

Culture, experience and learning all impact food preferences, but genetic factors can also play a role in evaluating food. For example, genetic variation in the bitter receptor T2R38 determines sensitivity to phenylthiocarbamide (PTC) [1], affects the taste of food containing bitter-tasting toxins and correlates with food preferences [2]. In addition to taste, odour is a major sensory component in flavour evaluation, yet how genetic variation in ORs affects food preferences remains unclear. It has been challenging to address because there are ∼400 human OR genes and hundreds of volatile chemicals found in various foods including meat [3], [4].

Androstenone, a steroid structurally related to testosterone, is a known pheromone in boars [5]. Androstenone, in combination with skatole, makes up the primary component of boar taint, an unpleasant odour and flavour found in pork derived from male pigs [6]. Skatole is a metabolite [7] of the amino acid tryptophan produced in the lower gut by the intestinal bacterial flora and has a faecal odour. Approximately ninety-nine percent of consumers have the ability to perceive skatole [8], and the compound can be detected in concentrations as low as 0.1 ppm [9]–[11]. Androstenone occurs in pork from male pigs in the range of 0–6.4 ppm. Although castration reduces the amount of androstenone in pork, the European Union recently proposed to ban castration due to animal welfare concerns [12]. This has reinvigorated the study of consumer perception of pork containing androstenone.

Unlike skatole, perception of androstenone varies from person to person, with descriptions ranging from urine and sweat to vanilla and sweet [13], [14]. While some subjects are insensitive to androstenone, others are highly sensitive and will react negatively upon exposure [15]. Androstenone in meat has been associated with flavours described as urine-like, etching (ammonia), pungent and sour [6], [16].

A recent survey showed that 39% of Norwegian consumers were identified as androstenone-sensitive, with negative reactions to meat containing higher levels of androstenone [11]. The fraction of androstenone-sensitive consumers in a population is highly relevant, as this figure could relate to the impact of specified androstenone levels on consumers’ acceptance, providing a background for assessing economical consequences of sending meat from uncastrated males into the market.

The ability to perceive androstenone correlates strongly with genetic variation in the human odour receptor OR7D4 [17]. A cell-based screen using an expression library of human ORs identified OR7D4 as a major androstenone receptor. We refer to the most common allele of this receptor, or the reference sequence, as OR7D4 RT. The other common allele contains two non-synonymous single-nucleotide-polymorphisms (SNPs) in complete linkage disequilibrium, resulting in two amino acid substitutions (R88W, T133M). We refer to this receptor as OR7D4 WM.

In cell-based assays, OR7D4 RT responds to androstenone while OR7D4 WM shows diminished responses. In a previous study, subjects with OR7D4 RT/WM and WM/WM genotypes were less sensitive to androstenone and found the odour less unpleasant than the subjects with the RT/RT genotype [17]. However, it is not known whether OR7D4 affects flavour perception of food containing androstenone such as pork.

Repeated exposure to androstenone induces increased sensitivity to androstenone, but only in about half of the exposed subjects [18]–[22]. Understanding how sensitivity to androstenone changes with respect to OR7D4 genotype may help us understand the mechanisms underlying the perception of and sensitization to androstenone, as well as estimate consumer acceptance of meat with boar taint.

The aim of the present study was to compare the ability of both the smell test used by Lunde et al [11] and OR7D4 genotype to predict perception of cooked meat samples containing different levels of androstenone.

We confirmed that OR7D4 genotype predicts sensitivity to androstenone. All the subjects who are classified as sensitive to isolated androstenone possessed two copies of functional OR7D4 RT alleles. Furthermore, our data suggests that OR7D4 genotype influences the evaluation of androstenone-tainted cooked meat samples.

## Results

### OR7D4 Genotype Predicts Androstenone Sensitivity

**Figure 1:**
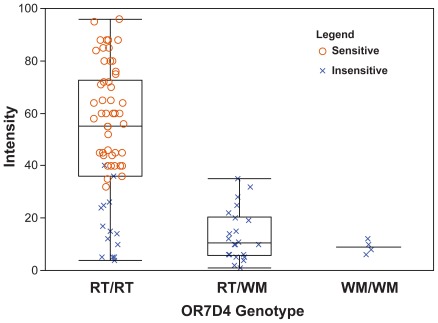
Genotypic variation in OR7D4 accounts for 40% of the variance in androstenone intensity. Subjects identified as sensitive to androstenone by the 2-trial 3AFC test are represented by circles, and subjects identified as insensitive are represented by Xs. Each subject rated the intensity of androstenone four times–all four ratings are plotted. Note that none of the subjects classified as sensitive carry the WM allele.

The subjects’ ability to detect androstenone as well as their intensity ratings were tested and correlated with their OR7D4 genotype. When subjects (naïve consumer subjects and trained assessors) were divided into sensitive and insensitive cohorts according to the smell test of Lunde et al. [11], we found that all androstenone-sensitive subjects had the RT/RT genotype. Four of the sixteen subjects with the RT/RT genotype were classified as androstenone-insensitive. Conversely all subjects with at least one copy of the nonfunctional WM allele were classified as androstenone-insensitive. The OR7D4 genotype explained 83% of the androstenone sensitivity classification (Fisher’s exact test, p<0.0013) and 40% of the variation in intensity ratings (ANOVA, F(2,85) = 29.0, p<0.0001, r^2^ = 0.40). The ability of both consumers and assessors to detect androstenone was correlated with OR7D4 genotype when analyzed separately (Consumer only (F(2,45) = 12.59, p<0.0001, r^2^ =  0.36). Assessor only (F(1,38) = 32.7, p<0.0001, r^2^ = 0.46). These data are consistent with the previously published findings [17] and confirm the role of OR7D4 in olfactory sensitivity to androstenone (Figure 1).

**Figure 2:**
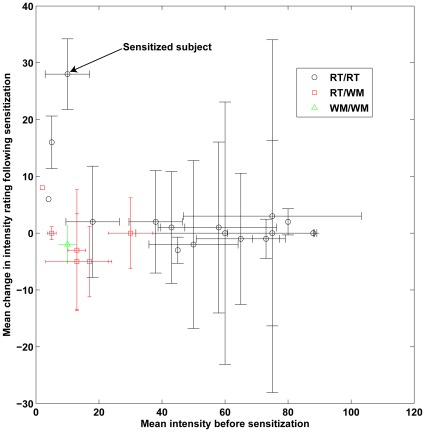
Change in intensity ratings following sensitization. The y-axis represents the mean of all possible pairings of ratings before sensitization with ratings after sensitization. Error bars represent standard deviation.

In prior studies, a portion of subjects were sensitized to androstenone after repeated exposure to the chemical [18]–[21]. The subjects’ sensitivity to androstenone was therefore compared before and after daily exposure to androstenone over a period of six weeks. Although as a group there was no significant difference between intensity ratings before and after sensitization (Wilcoxon, Z = 0.35, p = 0.72), one RT/RT subject who was initially classified as androstenone-insensitive was reclassified as sensitive using the smell test after the sensitization period. As a result, OR7D4 genotype explained the intensity of androstenone after sensitization better than the intensity of androstenone at the initial screening (Figure 2). The low number of subjects showing sensitization precludes us from drawing any significant conclusions.

### OR7D4 Genotype Predicts Acceptance of Meat Containing Androstenone

The next question was whether OR7D4 genotype correlated with the perception of cooked meat samples tainted with androstenone. Synthetic androstenone was added in the samples evaluated in this study so that androstenone sensitivity could be studied independent of variations in skatole concentrations and other compounds found in pork. This is important given that small amounts of skatole can influence the analysis and that skatole can be detected at levels as low as 0.1 ppm [9]–[11]. In addition, the variation in the samples presented to the subjects was minimized as all samples contained the same amount of fat, skatole and androstenone (Table 1).

**Table 1:** The androstenone and skatole content of the boar-tainted samples evaluated in this study.

Sample Name	Androstenone content (ppm)	Skatole content (ppm)
Reference	0	≤0.05
A3	3	≤0.05
A3.7	3.7	≤0.05
A4.5	4.5	≤0.05
A5.2	5.2	≤0.05
A6	6	≤0.05
A7.5	7.5	≤0.05

The androstenone values were measured in fat. All samples had 20% fat content.

The quality of synthetic skatole and androstenone was also measured. The samples were compared to biological compounds using NMR and were found to be 99.9% pure. The skatole and androstenone values referred to in this text were values measured in fat (not fatty tissue), and the levels are presented in Table 1. The levels of androstenone were within the naturally-occurring range.

#### Consumer testing

To test the effect of OR7D4 genotype on cooked meat preference containing androstenone, we first tested naïve consumer subjects for their odour perception (presumably orthonasal olfactory perception) and flavour perception (presumably taste and retronasal olfactory perception) of the samples. Consumers as a group tended to dislike cooked meat flavour containing more androstenone; an ordinal logistic regression showed that consumer evaluations predicted the androstenone content of the samples when rating the flavour (chi square = 6.07, df = 1, p<0.014, after Bonferroni correction p<0.042), but not the odour (during frying, chi square = 1.65, df = 1, p = 0.20; finished, chi square = 1.10, df = 1, p = 0.29).

**Figure 3:**
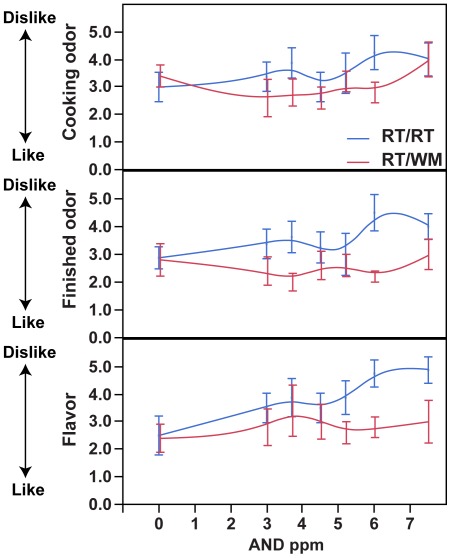
Consumer evaluation of cooked meat samples. Error bars represent standard error and lines represent a smoothing spline. Note that the scores are inverted for easier comparison with Figure 4. On this figure a rating of 1 indicates “like very much” and a rating of 7 indicates “dislike very much”.

When the subjects were divided by OR7D4 genotypes, there was a genotype effect on consumer preference. RT/RT subjects disliked the flavour and odour of the finished samples more than the WM carriers, but not the odour during frying (flavour, chi square = 10.12, df = 1, p<0.001, after correction p<0.003; finished, chi square = 9.24, df = 1, p<0.002, after correction, p<0.006; during frying, chi square = 1.45, df = 1, p = 0.23) (Figure 3).

Four of the subjects classified as insensitive to androstenone had the RT/RT genotype. One of these subjects was classified as sensitive after six weeks of daily exposure to androstenone. This subject gave low liking scores for androstenone after the sensitization experiment, consistent with the observation that this subject had been sensitized. However, the low number of subjects showing sensitization precludes us from drawing any significant conclusions.

#### Assessor testing

**Figure 4:**
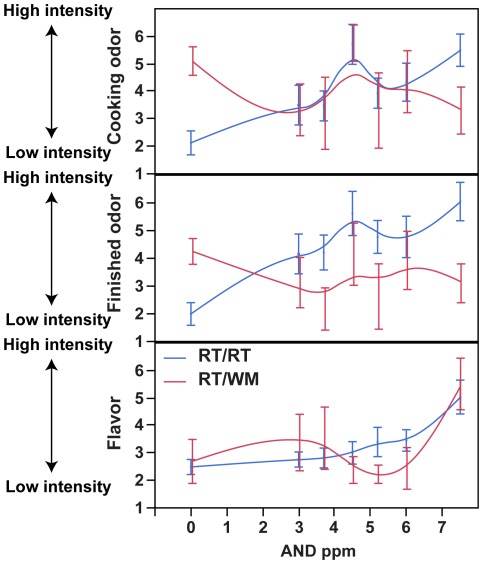
Sensory assessor evaluation of cooked meat samples. Error bars represent standard error and lines represent a smoothing spline. A rating of 1 was labelled “low intensity” and a rating of 9 was labelled “high intensity”.

Trained assessors are widely used in evaluating meat samples. To test OR7D4 genotype effects on cooked meat evaluation containing androstenone, we trained and tested assessors with cooked meat samples containing androstenone (see Materials and Methods for details). An ordinal logistic regression showed that the assessors’ androstenone intensity evaluations predicted the androstenone content of the samples when rating the flavour, but not the odour (flavour, chi square = 8.16, df = 1, p<0.0043, after correction p<0.013; finished, chi square = 3.85, df = 1, p = 0.05, after correction p = 0.15; during frying, chi square = 2.21, df = 1, p = 0.14) (Figure 4). There was a significant interaction between androstenone concentration and genotype for both odour evaluations (during frying, chi square = 6.56, df = 1, p<0.01, after correction p<0.03; finished, chi square = 7.47, df = 1, p<0.006, after correction, p<0.018), reflecting the observation that subjects with the WM allele did not increase their intensity evaluations with androstenone content. However, assessors with the WM allele gave flavour ratings that varied with the androstenone content of the samples and there was no interaction effect (chi square = 0.05, df = 1, p = 0.83). This may be related to the high androstenone concentrations in the meat containing 7.5 ppm androstenone. Though future research is necessary to confirm, this finding raises the possibility that people with the WM allele can be trained to evaluate androstenone flavour, but not odour, in meat samples. It is also possible that subjects with the WM alleles have a much higher threshold for odour.

## Discussion

The OR7D4 genotype explained 40% of the variation in intensity ratings in Norwegian subjects used in this study. This number is similar to previously published data with subjects in New York City [17]. These studies together strongly indicate that OR7D4 has a major role in perceiving androstenone. The remaining 60% of the variation may be explained by other ORs, other genetic factors, and non-genetic factors such as test variations, learning and culture.

A portion of the population is known to show dramatically increased sensitivity to androstenone after repeated exposure to androstenone [18]–[21]. Consistent with this notion, one of the subjects in our study was classified as sensitive after six weeks of daily exposure to androstenone. This subject with RT/RT genotype gave low liking scores for androstenone after the sensitization experiment, consistent with the observation that this subject had been sensitized. It is tempting to hypothesize that RT/RT subjects are more likely to be sensitized than subjects with WM, but more subjects will need to be tested.

Consumers in many developed countries have not experienced androstenone-containing meat since there has not been meat production from intact males for years. The data raise the possibility that more consumers will dislike male meat as a result of a castration ban.

What is the implication of this study in human genetics and dietary selection? It is tempting to speculate that certain ORs or variants of ORs influence dietary selection. These ORs might be selected during human evolution based on the available food source in a given habitat. For example, OR7D4WM might be concentrated in population that consume pork as a primary meat source because people with OR7D4WM might have found pork more attractive than those with RT/RT. It would be intersting to ask whether frequency of OR7D4 and other OR alleles correlate with pork and other food preference in different ethnic groups. In addition, future experiments with increasing subject numbers from different ethnic/cultural group would add more power to the study and minimize cultural biases of food selection.

Our data raise the possibility that the detection of androstenone flavour in the mouth was more sensitive than the detection of the androstenone odour by sniffing; this is consistent with the results from the evaluation and previously published results [16], but the cause is unclear. Androstenone may be vaporized more efficiently in the mouth when evaluating flavour. Relatively high temperature of the mouth might be a factor, as assessors could not discriminate samples of cold ham containing 3.96 ppm androstenone from control samples (K. Lunde, unpublished.) Alternatively, other volatiles might mask androstenone odour when smelling. Another possibility is that humans might be more sensitive to androstenone when sensing retronasally. These possibilities are not mutually exclusive and future study is necessary to address these issues.

This work is the first to link a simple smell test without false positive results regarding genotype, since all the subjects classified as sensitive have two copies of RT variant. Though the false positive rate will not likely be zero in a larger cohort, the smell test will be useful with respect to recruiting assessors with RT/RT to sensory panels without genetic testing. In the future, establishing sensory panels of specific genotypes can become highly economical as the standard deviation of attributes to be evaluated may become reduced. In addition, assessment of the size of market segments could become more accurate when combined with genetic polymorphism information of the population of interest.

Our study raises the possibility that a person with proper genotype (i.e. OR7D4 RT/RT) and the right threshold can be selected for screening in the slaughterhouse for eliminating meat with high concentration of androstenone. Heating the samples could greatly enhance the detectability of androstenone. It may be difficult for the evaluator to perform consistently due to changes in sensitivity over time. On one hand, adaptation may reduce the sensitivity and on the other the tester may be sensitized over time. Nevertheless, the idea that a qualified person could serve as a grader of androstenone tainted meat should now be straightforward to test.

In conclusion, the results showed that OR7D4 genotype correlated with androstenone sensitivity as well as the subject’s perception of cooked meat samples containing androstenone. Our study suggests that functional variation in an OR can alter food preferences. Further work is needed to understand how an individual’s unique OR repertoire contributes to overall flavour evaluation and preference of meat and other foods.

## Materials and Methods

### Ethics Statement

The participants were informed about the project and procedures according to instructions from The National Committees for Research Ethics in Norway. The participants were able to drop out at any time during the study without consequence. Approvals to collect, export and analyse the DNA of recruited subjects were given by the Regional Committees for Medical Research Ethics in Norway, the Norwegian Directorate for Health and the Norwegian Social Science Data Services.

### Recruitment of Subjects

Subjects for this study were recruited following sensitivity testing in Norway [11]. All subjects gave consent to participate, and were financially compensated for their time and efforts. A total of 23 subjects were recruited: 13 consumers and 10 professional sensory assessors.

### Sampling of Blood, Isolation of DNA and DNA Typing

Trained health care personnel collected the blood samples and DNA was isolated at the Norwegian University of Life Science using the method described by Keller et al. [17]. For sequencing, human genomic DNA was amplified with HotStar Taq (Qiagen) with primers upstream (5′AAGTGATGACAAGCTGAGCTGC-3′) and downstream (5′CCACAACATTTGCCTTAGGGGTA-3′) of the OR7D4 open reading frame. The PCR products were then Sephacryl S-400-purified (GE HealthCare) and sequenced with a 3100 or 3730 Genetic Analyzer (ABI Biosystems).

### Androstenone Sensitivity Among Participating Subjects

The subjects participating in this study were selected among subjects who were previously tested for their ability to perceive androstenone through a smell test [11] in a large screening of androstenone sensitivity done in Norway in 2008 [15]. We tested only orthonasal odour perception. The smell test involved the intensity rating of androstenone crystals in water in a double 3-Alternative Forced Choice (AFC) test. In each of the 3 AFC tests, two bottles with water and one bottle with androstenone were presented and the subject chose the sample with the strongest odour. This scale was anchored with “barely detectable” at the lower end and “strongest imaginable” at the higher end. The qualitative intensity scale was converted to a quantitative one from 0 to 100. Twelve sensitive and eleven non-sensitive subjects were selected for further testing.

### Androstenone Sensitization with Time

All subjects participating in this study were exposed to androstenone daily for six weeks after the initial testing. The sensitization experiment was performed after the evaluation of meat samples (see below) in all cases except one. The androstenone solution used in the sensitization experiment was the same as the solution used in the sensitivity test (0.0017 g androstenone crystals added to 10 ml water). This amount ensures that the water was saturated with androstenone for an extended period. The subjects were told to store the bottle at room temperature and to sniff the bottle immediately after taking the cap off once daily.

### Evaluation of Meat Samples

The subjects evaluated cooked meat samples with different levels of androstenone. In this study, seven samples of minced meat with different levels of androstenone were evaluated. Fat from different castrates with skatole levels at ≤0.05 ppm (skatole is naturally present among castrates in Norway at an average level of 0.07 ppm, but samples that had ≤ 0.05 ppm skatole) were mixed with synthetic androstenone (5α-androst-16-en-3-one) from Sigma–Aldrich, Co Ltd (Poole) dissolved in 10 ml ethanol.

The fat tissue was mixed with meat from *Semimembranous* muscle according to the experimental design shown in Table 1. Sample preparation was done at Nofima Mat in Norway, and is described in detail by Lunde et al. [16]. 1% water and 1% salt were added to each batch. Samples (50 g) with a thickness of approximately 2 mm and a diameter of approximately 15 cm were made by hand, then vacuum-packed and kept frozen (−20°C). The samples were similar to a product already produced in the Norwegian market. The subjects were requested to keep the samples frozen until they were fried.

### Instrumental Measurements of Skatole and Androstenone

Skatole and androstenone values were measured in the fat mixtures before processing. Skatole was determined in extracted fat by HPLC (Agilent Technologies) using fluorescence detection according to a method developed by Gibis [23]. The androstenone content was determined by a time-resolved fluorescent immunoassay as described by Tuomola et.al. [24], modified using antiserum produced and characterized by Andresen [25].

Synthetic skatole and androstenone were compared to the biological compounds using NMR spectra. NMR spectra were recorded in CDCl_3_ using the solvent as the reference set at 7.24 for the ^1^H NMR and 77.23 for the ^13^C NMR values.

### Consumer Testing

The samples (minced meat) with different levels of androstenone (Table 1) were fried in a preheated frying pan and evaluated by 13 consumers in a home test during a period of several days. If more than one sample was evaluated during a day, the consumers were instructed to have at least a one-hour break while ventilating the room before evaluating the next sample. Between each sample, the consumers were told to clean the frying pan with soap and rinse thoroughly. Liking of odour during frying, liking of odour of the fried meat, and liking of flavour during eating were evaluated on a seven point scale with “dislike very much” rated as a “1” and “like very much” rated as a “7”. In addition, the consumers were allowed to comment on each sample.

The samples were evaluated in the order they appeared in on the questionnaire, which was randomized for each subject. The samples were evaluated before the sensitization experiment in all cases except for one subject whose results were obtained after becoming sensitive through the six-week sensitization period. The subjects were classified as sensitive or insensitive by the method described by Lunde et al. [11].

### Sensory Analysis by Assessors

The sensory analysis was performed by the sensory panel at Nofima Mat in Norway. The panel consisted of 10 trained (7 sensitive) assessors with 4 to 20 years of general experience in sensory profiling. The panel has had several years of experience evaluating boar-tainted meat, especially during the last 5 years. The samples were evaluated in a sensory laboratory designed according to guidelines in ISO (1988) with separate booths and electronic registration of sensory data.

### Sensory Profile

The profile used was the same as the profile used in the study with four sensory panels across Europe [16]. The profile consisted of the attributes skatole (intensity of skatole), androstenone (intensity of androstenone) and rancid (intensity of all rancid odours–grass, hay, paint, stearine). Rancid was included as an attribute in the profile since rancidity is one of the more common off-flavours in pork meat.

### Training of Assessors

The sensory assessors were experienced in the evaluation of boar-tainted meat, and were recently trained on boar-tainted meat samples. The training of assessors was therefore done using three samples: one reference sample (no androstenone or skatole added), one sample with high androstenone content (7.5 ppm) and one sample with high skatole content (9.0 ppm). The androstenone level in the training samples corresponded to the highest androstenone level of samples in the experiment. The samples were evaluated on a 9 point unstructured continuous scale, where a “1” corresponded to “low intensity” and a “9” corresponded to “high intensity”. The assessors were trained using the attributes in the profile. Training included perception of the attributes during frying (only odour) and evaluation in the booth (odour and flavour).

### Sensory Analysis of Boar Tainted Samples

The assessors evaluated the odour of the sample above the frying pan both during and following frying. They then evaluated the flavour of the finished sample by consuming the meat. The assessors evaluated the rancidity of the meat as well as the intensity of skatole and androstenone. The same attributes were used for both odour and flavour evaluation.

The frozen samples were fried in neutral oil in a pre-heated pan with lid. The samples evaluated in the frying pan (odour) were divided in 5 parts (approx. 10 g each) before frying. The samples were fried in a pan covered with a lid for 1 minute before the lid was taken off and the assessors then sniffed the samples one by one while still frying them. The frying pan was cleaned with soap and rinsed thoroughly between each sample.

Samples evaluated in the booth (odour and flavour) were fried in a warm pan with a lid on top for approximately 1 minute on each side until the meat was well-done. The assessors divided the samples into approximately 25 g portions before frying. The samples were served at a temperature of 60°C in boxes suitable for taste analysis with a lid. The assessors evaluated odour after taking the lid off, and then flavour while eating. The assessors rinsed their mouths with water and/or some neutral crackers between the samples.

The samples were served in a randomized order. Odour assessments during frying and odour and flavour assessments after frying were run in different sessions, with a break (30 minutes) between sessions.

### Statistical Analysis

Statistical analyses were performed in JMP 9 (SAS Institute Inc., Cary, NC, USA). The data in Figure 3 were analyzed using three separate ordinal logistic regression models (one each for flavour, finished odour, and cooking odour) with model effects of sample androstenone concentration, OR7D4 genotype and their interaction. Ratings of flavour, finished odour, and cooking odour were treated as ordinal variables. The effects were tested using likelihood ratio tests and the alpha value was bonferroni-corrected to account for the three separate tests. Similarly, the data in Figure 4 were analyzed using three separate ordinal logistic regression models (one each for flavour, finished odour, and cooking odour) with model effects of sample androstenone concentration, OR7D4 genotype and their interaction. The effects were tested using likelihood ratio tests and the alpha value was bonferroni-corrected to account for the three separate tests.
